# Study on Interactions between the Major Apple Valsa Canker Pathogen *Valsa mali* and Its Biocontrol Agent *Saccharothrix yanglingensis* Hhs.015 Using RT-qPCR

**DOI:** 10.1371/journal.pone.0162174

**Published:** 2016-09-09

**Authors:** Dongying Fan, Yanfang Li, Lingyun Zhao, Zhengpeng Li, Lili Huang, Xia Yan

**Affiliations:** 1 State Key Laboratory of Crop Stress Biology for Arid Areas, Northwest A&F University, Yangling, Shaanxi, China; 2 College of Life Science, Northwest A&F University, Yangling, Shaanxi, China; 3 College of Plant Protection, Northwest A&F University, Yangling, Shaanxi, China; Gyeongnam National University of Science and Technology, REPUBLIC OF KOREA

## Abstract

The mechanism of biocontrol agent *Saccharothrix yanglingensis* Hhs.015 action against *Valsa mali*, a major apple Valsa canker pathogen, was examined using a novel, sensitive (minimum detection limit 100 pg/μL) and reliably RT-qPCR technique. Prior to lesion formation, total concentration of *V*. *mali* in the bark showed a significant decrease (*p*<0.05) after 24 h of Hhs.015 treatment. This was more pronounced at 48 and 96 h post treatment. After lesion formation, levels of *V*. *mali* remained constant at the boundary between infected and uninfected bark tissues, although the relative expansion rate of the lesion was significantly reduced (*p*<0.05). Gene expression levels of endo-polygalacturonase, a marker for fungal pathogenicity, were sharply reduced while host induced resistance callose synthase levels increased significantly (*p*<0.05) at the boundary bark at 9 d after Hhs.015 treatment. The results showed that biocontrol agent Hhs.015 prevented infection of *V*. *mali* by inhibiting pathogen growth, down-regulating pathogenicity factor expression and inducing a high level of host resistance.

## Introduction

Apple Valsa canker is a serious fungal disease caused predominantly by *Valsa mali* [1], which impacts apple productivity and produces huge economic losses in East Asia, especially China [2]. The causal agent can induce elongated lesions on the tree trunks and limbs, and eventually produce death of the tree followed by possible destruction of the whole orchard. A number of cell wall-degrading enzymes and toxins are involved in the pathogenesis of apple Valsa canker [3–4]. However, effective prevention and control systems for this disease are still unavailable [5].

Currently, the strategy primarily used to control apple Valsa canker involves scraping the lesion then spreading on fungicides [6]. However, the effectiveness of the current control measure has proven unsatisfactory primarily because the pathogen can penetrate deeply into both the xylem and phloem of the host [7]. Furthermore, chemical approaches have led to environmental and health risks [8]. Biological control are used to control plant diseases because they are environment-friendly and maintain sustained effectiveness [9]. Identification of effective biocontrol agents has previously focused on isolation of soil microorganisms, but this has generated an increasing number of problems [10]. More recently, microorganisms in plants, namely endophytes, have provided a potential source of biocontrol agents [11]. Among the endophytes, actinomycetes has been shown to be capable of producing antibiotics and exoenzymes appear promising for controlling a variety of different plant diseases [12].

*Saccharothrix yanglingensis* Hhs.015 is a novel endophytic actinomycete isolated from cucumber roots [13], that has shown efficacy against apple Valsa canker in both indoor and field trials. When treated with fermentation broth of Hhs.015, the germination rate of *V*. *mali* conidia is sharply reduced. Morphologically, the fungal mycelium became crimped while the tips of hyphae were exceedingly ramified. Field trials showed that the recurrence rate of scraped lesions in *V*. *mali*- infected apple trees was stably controlled by Hhs.015 with a high level of efficiency (61.29%), which was comparable to common chemical agents such as difenoconazole and tebuconazole [14]. Early studies have shown that Hhs.015 produced heteroauxin, chitinase, proteinase and glucanase. Some active substances were purified from fermentation broth of Hhs.015, such as isoflavones and Pentamycin [13].

Conventionally, biocontrol agents effects are assessed via *in vitro* growth inhibition tests and/or by visual quantification of lesion growth rate on tree limbs. Both methods are time-consuming and subjective, especially when a large number of samples are to be tested [15]. Other methods have been reported, such as enzyme-linked immunosorbent assays for measuring fungal-specific constituents (e.g., chitin and ergosterol) [16] using a fluorescent green protein reporter gene for monitoring pathogenic fungi, and a sequence-based methods such as RNA hybridization and semi-quantitative PCR [17]. However, none of the above methods can simultaneously quantify pathogen levels and monitor pathogenicity factors. It is necessary to develop a new approach that can directly measure the amount of the pathogen present in host plants even in the absence of disease symptoms. The development of quantitative real-time PCR (RT-qPCR) technique has been an important step toward quantative analysis since its first application for the detection of *Phytophthora* strains in their host plants [18], the RT-qPCR approach has gained acceptance based on its high level of efficiency, sensitivity, reliability, reproducibility and robustness [19]. Moreover, the RT-qPCR assay permits simultaneous evaluation of gene expression of multiple pathogenicity and host-resistance factors [20]. As a housekeeping gene, the glucose 6-phosphate dehydrogenase (G6PDH) gene is stably expressed in *V*. *mali* under select experimental conditions [21]. Any quantitative changes in *G6PDH* gene expression reflects corresponding quantitative changes of *V*. *mali* before or after bio-control treatment. It has been determined that *V*. *mali* pathogenesis involves cell wall degrading enzymes and toxins [3–4]. Pectinases, such as endo-polygalacturonase (EPG) are considered to be key pathogenicity factors in fungi [22]. Moreover, fungal infection can cause morphological and structural changes such as formation of a callose in the host [23] leading to resistance against fungal pathogens.

The purpose of the present study was to elucidate the biocontrol mechanisms of Hhs.015 against *V*. *mali* based on antifungal activity and host induced resistance. A novel, specific *G6PDH*-based RT-qPCR method was developed to assess quantitative changes of *V*. *mali* as well as the gene expression levels of *EPG* and callose synthases (*CALS*) before and after treatment with Hhs.015. The results will provide new insights into the biocontrol mechanisms of the endophytic *S*. *yanglingensis* Hhs.015 against apple Valsa canker.

## Materials and Methods

### Fungal strain, endophytic actinomycete and plant material

A highly pathogenic isolate of *V*. *mali* (03–8) and the endophytic actinomycete *Saccharothrix yanglingensis* Hhs.015 were provided by the Laboratory of Integrated Management of Plant Diseases, College of Plant Protection, Northwest A&F University, Yangling, Shaanxi Province, China. Two-year old apple twigs (*Malus × domestica* cv. Fuji) were obtained from healthy trees grown in an apple orchard in Jiaosheng Village, Yangling (the study was carried out on private land, and the owner of the land gave permission to conduct the study on this site).

### Inoculum preparation and plant inoculation

The fungal isolate 03–8 was cultured on potato dextrose agar (PDA) plates and incubated at 25°C for 4 d. Inoculation of 03–8 to tree twigs was performed following the procedure described previously [4]. Twigs of the same size (diameter 10 mm) were cut into 30 cm length, washed with tap water, and then immersed in 0.6% sodium hypochlorite for 30 min. After being rinsed with sterile water three times and air dried, both ends of the twigs were sealed with paraffin wax. Five-mm-diameter agar plugs were aseptically cut out from the edge of fungal colonies grown on PDA medium. The plugs were placed next to the scorch sites and covered with a moisturizing piece of wet sterile absorbent cotton. The inoculation site was immediately covered with a vinyl film and twigs were placed into sand pots (internal diameter 17.5 cm) to maintain the humidity at 25°C.

Strain Hhs.015 was cultured on Gause's No 1 synthetic agar medium plates at 28°C for 7 d [13]. Eight 5-mm-diameter agar plugs were cut out and inoculated into a 250-mL flask containing 100 mL of tryptic soy broth medium (NaCl 5 g·L^-1^, tryptone 17 g·L^-1^, soy peptone 3 g·L^-1^, K_2_HPO_4_ 2.5 g·L^-1^, glucose 2.5 g·L^-1^, and pH 7.0). The liquid culture was incubated at 28°C, 150 rpm for 7 d. Fermentation broth of Hhs.015 was spread on the pathogen inoculation sites before or after appearance of lesions on twigs (1 or 4 d after inoculation of *V*. *mali*). The length of lesions was measured before sampling of bark tissue.

Prior to lesion formation, bark samples were collected from all of the inoculation sites at 0, 12, 24, 48, 72 and 96 h after treatment with Hhs.015. After lesions formation, bark at the boundary between diseased and healthy areas of twigs were collected 0, 1, 2, 5, 7, 9, 11 and 16 d later. All samples were frozen in liquid nitrogen and processed immediately for RNA extraction. The experiment was conducted in triplicate.

### RNA extraction and cDNA synthesis

Total RNA from all samples was extracted using the cetyltrimethyl ammonium bromide (CTAB) method [24] with slight modifications. A portion (~400 mg) of finely powdered sample ground in liquid nitrogen was added to an RNase free centrifuge tube containing preheated CTAB extraction buffer. The mixture was vortexed for 5min and incubated at 65°C for 30 min.

An equal volume of phenol-chloroform—isoamylalcohol (25:24:1) was added and extracted twice by centrifugation at 12000 *g* for 15 min at 4°C. The supernatant was recovered and combined with 1/3 volume of 8 M LiCl. After a 2 h precipitation period at -20°C, an RNA pellet was obtained by centrifugation at 12000 *g* for 30 min at 4°C. The pellet was resuspended in 70% ethanol and centrifuged at 12000 *g* for 15 min at 4°C followed by repeated ethanol extraction. The RNA extract was dissolved in 100 μL of DEPC-treated ddH_2_O, added with 1/10 volume of 3 M NaAc (pH 5.2) and three volumes of ethanol, then precipitated for 30 min at -20°C.

The RNA pellet was recovered by centrifugation at 12000 *g* for 30 min at 4°C, washed twice with precooled 70% ethanol, and centrifuged at 12000 *g* at 4°C for 10 min. The precipitated RNA was air-dried and resuspended in 20 μL of DEPC-treated ddH_2_O. The integrity of the RNA extract was evaluated by electrophoresis on a 1.0% agarose gel. Samples exhibiting an A260/A280 ratio of 1.8–2.1 and an A260/A230 ratio>2.0 were selected and stored at -80°C for further use.

First strand cDNA synthesis was performed in a final volume of 20 μL using a Revert Aid First Strand cDNA Synthesis Kit (Thermo Scientific Lithuania) and oligo-dT primers following the manufacturer’s instructions. Genomic DNA contamination in cDNA was checked by PCR as described previously [25]. All cDNA samples were stored at -20°C before use.

### Primer design and specificity testing

Primers for *V*. *mali G6PDH* (KC248180) and *EPG* were designed as previously described [21], and primers for elongation factor 1á (EF1) was designed according to sequence data of *Malus × domestica* cv. Fuji (DQ341381.1) [26]. Primers for *CALS1* and *CALS2* [27] were designed using Primer 5.0 (Premier Biosoft International, Palo Alto, CA, USA. Table 1). All primers were synthesized by Sangon Biotech, Shanghai, China.

**Table 1:** Primers for CALS1 and CALS2 used in this study.

Primers	GenBank accession no.	Nucleotide sequences (forward/reverse) [5′-3′]	Amplicon size (bp)	T_m_ (°C)
CALS1	FN395073	CAAAAAGTCCCAATGAGATGC	98	87.5
		AGAAGAGGAGGAAAGATGTGCT		
CALS2	FN395074	TTCACGGAAGAGTGGAGGATT	155	84.5
		TCAGATGCGGGAGGGTAAC		

To check the specificity of the primers, conventional end-point PCR was performed with a cDNA template of *V*. *mali* or healthy bark smeared with Hhs.015 fermentation broth only. All reactions were performed in a 25-μL volume containing 1 × reaction buffer, 1.5 mM MgCl_2_, 2.5 mM each dNTPs (Roche, Mannheim, Germany), 5 pM each primer, 0.4 U *Taq* DNA polymerase (Thermo Scientific, Lithuania), and 50 ng of cDNA. PCR amplification was carried out as follows: 95°C for 5 min followed by 34 cycles of 95°C for 30 s, 55°C for 30 s and 72°C for 45 s, and a final extension at 72°C for 10 min. The quantity of PCR products was analyzed on a 2% agarose gel.

### RT-qPCR assay

Experimental samples were analyzed using a Bio-Rad iQ5, qPCR assay in 25 μL reactions containing 1 μL of cDNA template, 0.4 μL of each primers (0.2 μM), 1 × reaction buffer, 2 2 μM MgCl_2_, 0.2 2 μM dNTPs (Roche, Mannheim, Germany), 0.4 U *Taq* DNA polymerase (Thermo Scientific, Lithuania), 2 μL of cDNA template, and 0.5 mL of 2 × SYBR (Takara, Tokyo, Japan). The PCR program was as follows: 95°C for 1 min, 40 cycles of 95°C for 10 s, 60°C for 10 s, and 72°C for 40 s. Finally, dissociation curves were generated by increasing the temperature from 65°C to 95°C.

To assess the effects derived from potential contamination of host cDNA on PCR detection, *V*. *mali* cDNA (1 ×10^3^ ng/μL) was serially diluted 1:10 to 1:10^7^ in *Malus × domestica* cv. Fuji cDNA (1 ×10^3^ ng/μL) or water. Three replicates of the dilution series were analyzed by RT-qPCR assay.

To determine the sensitivity and minimum detection limit of the RT-qPCR approach, standard curves for *G6PDH* and *EF1* were generated by plotting the log10-transformed cDNA concentration of known standards against the cycle threshold values (Ct) measured using CFX Manager Software (Bio-Rad Laboratories, Uercules, CA), and the range of the linearity was determined. These curves allowed for transformation of experimental Ct values into the amounts of cDNA and pathogen. All experiments were repeated three times and each dilution was assayed in triplicate. A no-template control (NTC) with water only was used as a negative control.

For intra-assay comparisons, a calibrator cDNA containing host cDNA at 1000 ng/μL and pathogen cDNA at 1 ng/μL was included in each experiment. This DNA was applied as a dual-species quantification standard by loading in triplicate in each assay.

### Quantitative determination and statistical analysis

The RT-qPCR assay was used to quantify *V*. *mali* in symptomatic and asymptomatic bark samples as described above. Each sample was amplified in three replicates. For quantification of the pathogens, a colonization coefficient (CC) method was used for data analysis [26]. The CC value of an Hhs.015-treated group was divided by that of a control group (T/C) to show the difference between treated and non-treated groups. A T/C ratio significantly greater than 1 indicates an increase in RNA levels from the Hhs.015 treatment and a T/C ratio less than 1 indicates a decrease in RNA levels from Hhs.015 treatment.

To assess relative gene expression levels of *EPG* and *CALS1* or *CALS2*, the Ct values were processed using the 2^ΔΔCt^ method with *G6PDH* and *EF1* as reference genes, respectively. Visual assessment of apple Valsa canker was measured by the relative expansion rate of the canker, that is, the length ratio between lesions of treated or control group and the initial sample at 0 h.

Data were analyzed using One-Way analysis of variance (ANOVA) according to the factorial design with three replications (IBM SPSS Statistics Version 20, Somers, NY, USA). Significance of differences between means was determined using the least significant difference test (*p*<0.05).

## Results

### RNA integrity and primer specificity

The integrity of total RNA isolated from bark samples was analyzed by agarose gel electrophoresis. Distinct 28S and 18S rRNA bands were observed (data not shown), indicating a high level of total RNA integrity. The A260/A230 and A260/A280 ratios for all RNA extracts were greater than 2.0 and within the 1.9–2.0 range respectively (data not shown).

Primers specificity was examined by electrophoresis of the PCR products obtained using cDNA template of *V*. *mali* or healthy bark smeared with Hhs.015 (NTC). PCR products of expected sizes were obtained for *G6PDH*, *EPG*, *EF1*, *CALS1*, and *CALS2* using their respective primers (S1 Fig). Melt curves of the RT-qPCR assay revealed a single dissociation peak at their melting temperatures, demonstrating the specificity of the amplification. Curves without a significant fluorescence increase or dissociation peak were obtained for NTC ruling out the possibility of primer dimer formation. Together, these results suggest that the primers used for RT-qPCR assays were specific for their respective genes.

### RT-qPCR sensitivity and specificity

**Fig 1:**
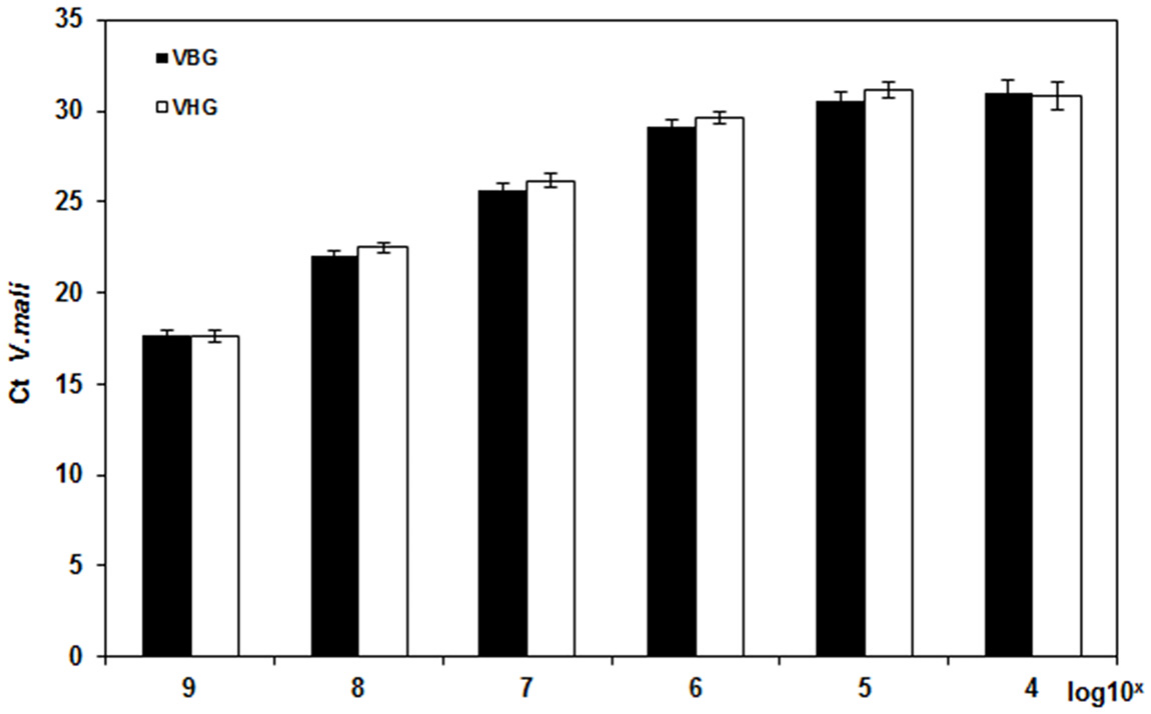
Threshold cycle (Ct) values of serially diluted *Valsa mali* cDNA (10^9^ to 10^2^ fg/μL) for *G6PDH*. *V*. *mali* cDNA was diluted in *Malus × domestica* cv. Fuji cDNA (10^9^ fg/μL; black bars) or in water (white bars). Bars represent the standard deviations. There were no significant differences between corresponding *Malus × domestica* cv. Fuji cDNA and water dilution classes at *p*>0.05.

To assess the specificity and sensitivity of RT-qPCR, *V*. *mali* cDNA was diluted one-to-ten in the presence or absence of host cDNA. The inclusion of plant-derived cDNA had no significant (*p*>0.05) effect on the quantification of *V*. *mali* cDNA (Fig 1).

**Fig 2:**
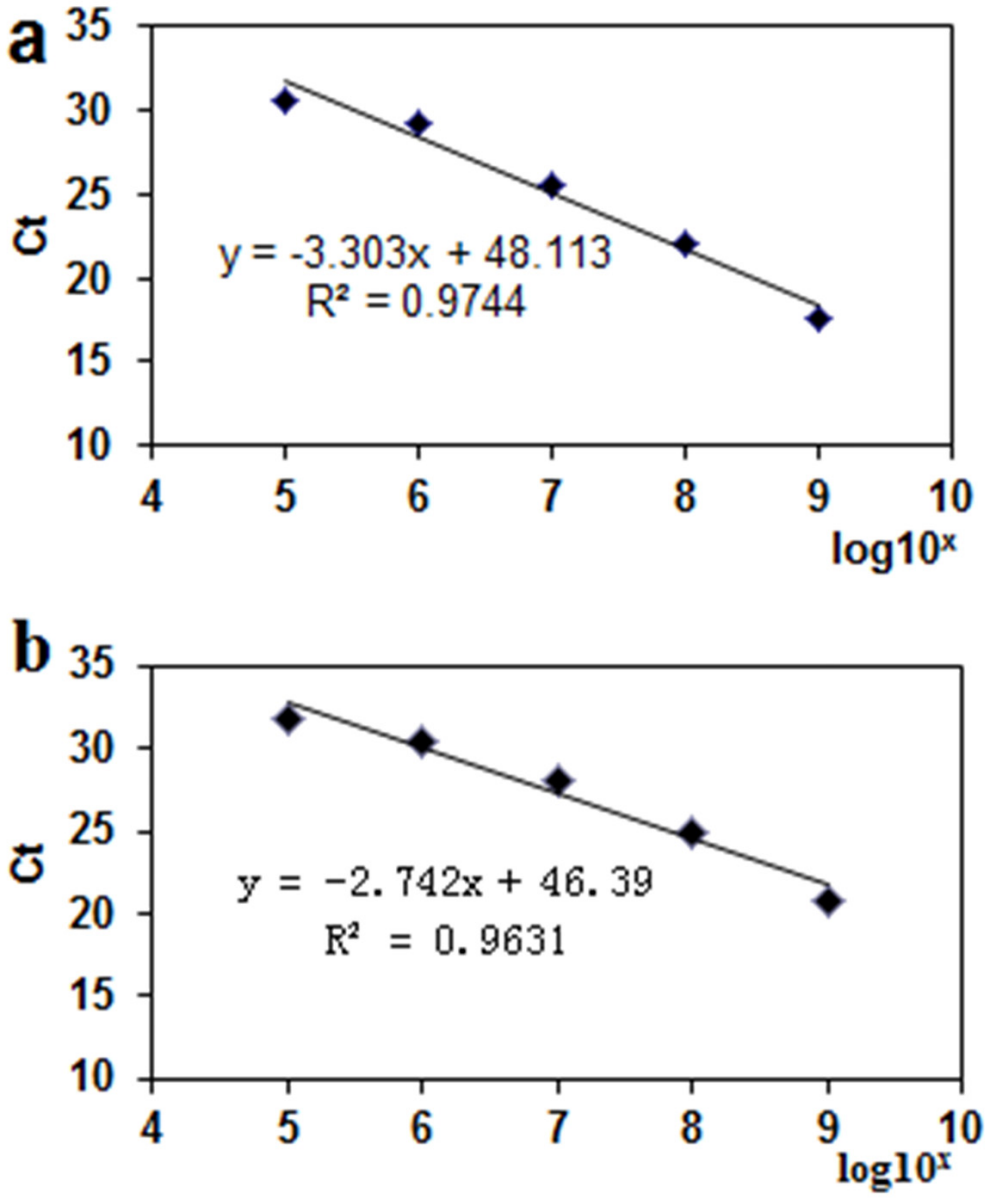
Standard curves for RT-qPCR assays of *G6PDH* and *EF1* mRNA expression. Standard curve for *G6PDH* (a) was generated using *V*. *mali* cDNA serially (10^9^ to 10^2^ fg/μL) diluted in host cDNA (10^9^ fg/μL). Standard curve for EF1 (b) was generated by *Malus × domestica* cv. Fuji cDNA diluted serially (10^9^ to 10^2^ fg/μL) in water. A linear regression curve was calculated for use to quantify RNA levels from plant samples.

Standard curves were generated for serially diluted *V*. *mali* cDNA in host cDNA (Fig 2a) and *Malus × domestica* cv. Fuji cDNA in ddH_2_O (Fig 2b). There was a strong correlation between the Ct values and log10-transformed amount of pathogen or host cDNA (*R*^*2*^ = 0.9744 and 0.9631, respectively), with linearity that extended over a 10^5^-fold range. The RT-qPCR assay failed to detect the amount of DNA at concentrations below 10^5^ fg/μL indicating a minimum detection limit of 100 pg/μL cDNA.

### Pathogen quantity as affected by Hhs.015

**Fig 3:**
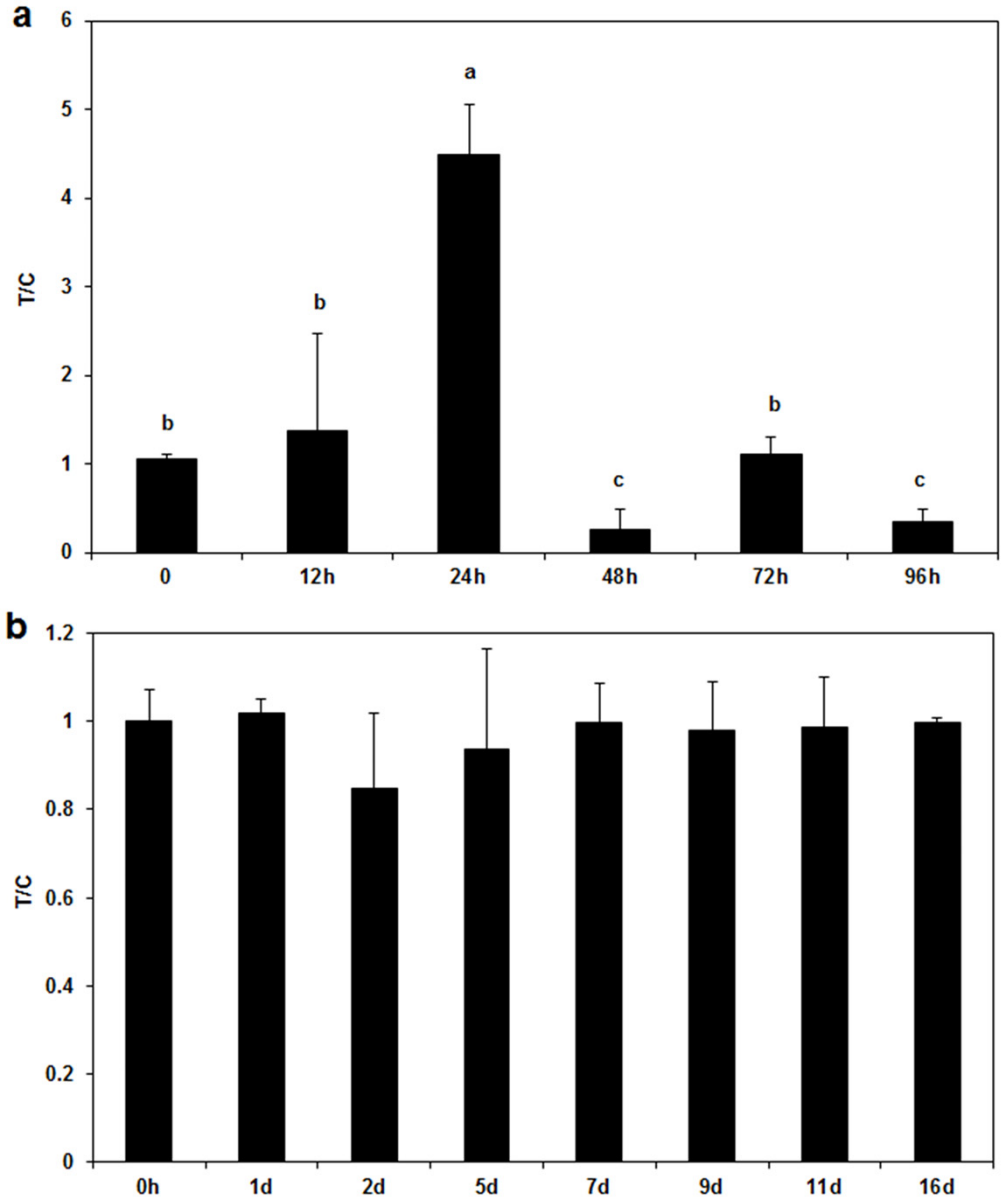
Quantification of V*alsa mali* in asymptomatic and symptomatic apple bark samples. Effects of application of Hhs.015 fermentation broth on *V*. *mali* in asymptomatic (a) and symptomatic apple bark samples (b) were assessed using the developed RT-qPCR method. T/C is a ratio of the colonization coefficient value of the treatment group over that of the control group. Bars represent the standard deviations. Each sample was amplified in three replicates. Statistical analysis was carried out using One-Way ANOVA. Different letters above the error bar (a) denote significant differences at *p*<0.05 for time factor.

To assess the applicability of the RT-qPCR method for detecting *V*. *mali* in plant tissues, infected but asymptomatic bark samples were first tested. After Hhs.015 application, there was an initial increase in the amount of the pathogen in asymptomatic bark samples, with a peak value at 24 h post treatment (*p*<0.05 for time factor) (Fig 3a). Thereafter, fungal growth was drastically inhibited since the T/C values significantly decreased (*p*<0.05 for time factor) at 48 and 96 h post treatment demonstrating a direct inhibitory effect of Hhs.015 on *V*. *mali*.

**Fig 4:**
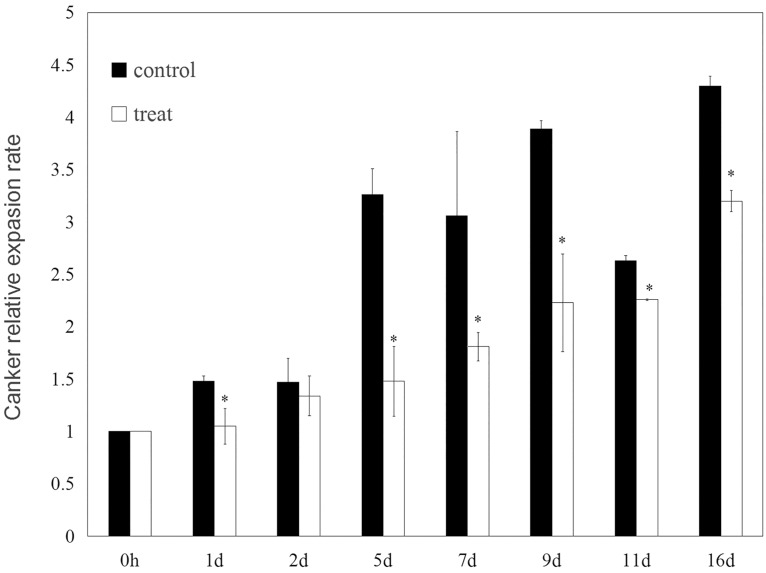
The relative expansion rate of canker in apple bark samples. The relative expansion rate of canker was calculated based on the ratio between the canker length and the initial twig length of the treatment or control group. Bars represent the standard deviations. Each sample was repeated three times. Statistical significance of the difference between means was evaluated using One-Way ANOVA. * denotes significant differences between the treatment and control groups (*p*<0.05 for treat factor).

Hhs.015 treatment did not affect the amount of pathogen present in bark at the boundary between diseased and healthy tissues after lesions development (P>0.05 for time factor) (Fig 3b). However, the presence of Hhs.015 led to a significant reduction (*p*<0.05 for treat factor) in the relative expansion rate of the lesion as compared with the control (Fig 4).

### Gene expression of pathogenicity and host resistance related factors affected by Hhs.015

To further understand the effect of Hhs.015 on *V*. *mali* infection, we investigated the gene expression profiles of pathogenicity and host resistance factors at the boundary between diseased and healthy tissues.

**Fig 5:**
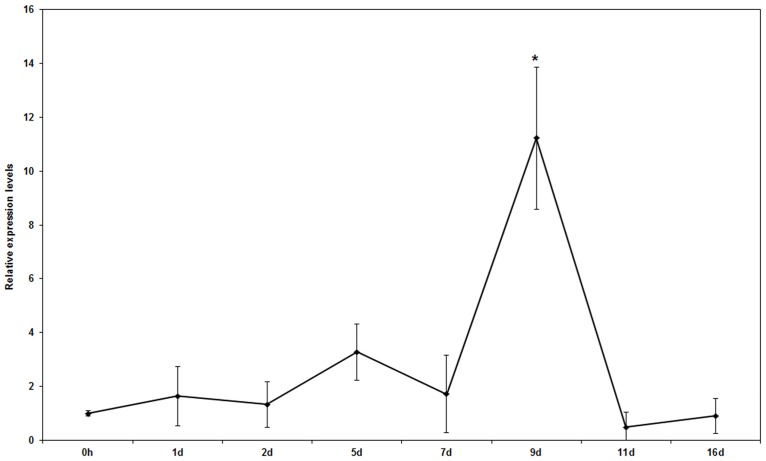
Effects of *Saccharothrix yanglingensis* Hhs.015 fermentation broth on *EPG* gene expression. Gene expression was assessed as described (M&M). Bars represent the standard deviations. Each sample was amplified three times. Statistical significance of the differences between means was evaluated using One-Way ANOVA. * denotes significant differences at *p*<0.05 for time factor.

The relative gene expression level of pathogenicity-related *EPG* remained unchanged at 7 d post treatment, then drastically increased at 9 d, after which it decreased continuously after 10 d (Fig 5, *p*<0.05 for time factor). These results suggest that Hhs.015 played a negative role in *V*. *mali* infection by down-regulating pathogenicity factor expression.

**Fig 6:**
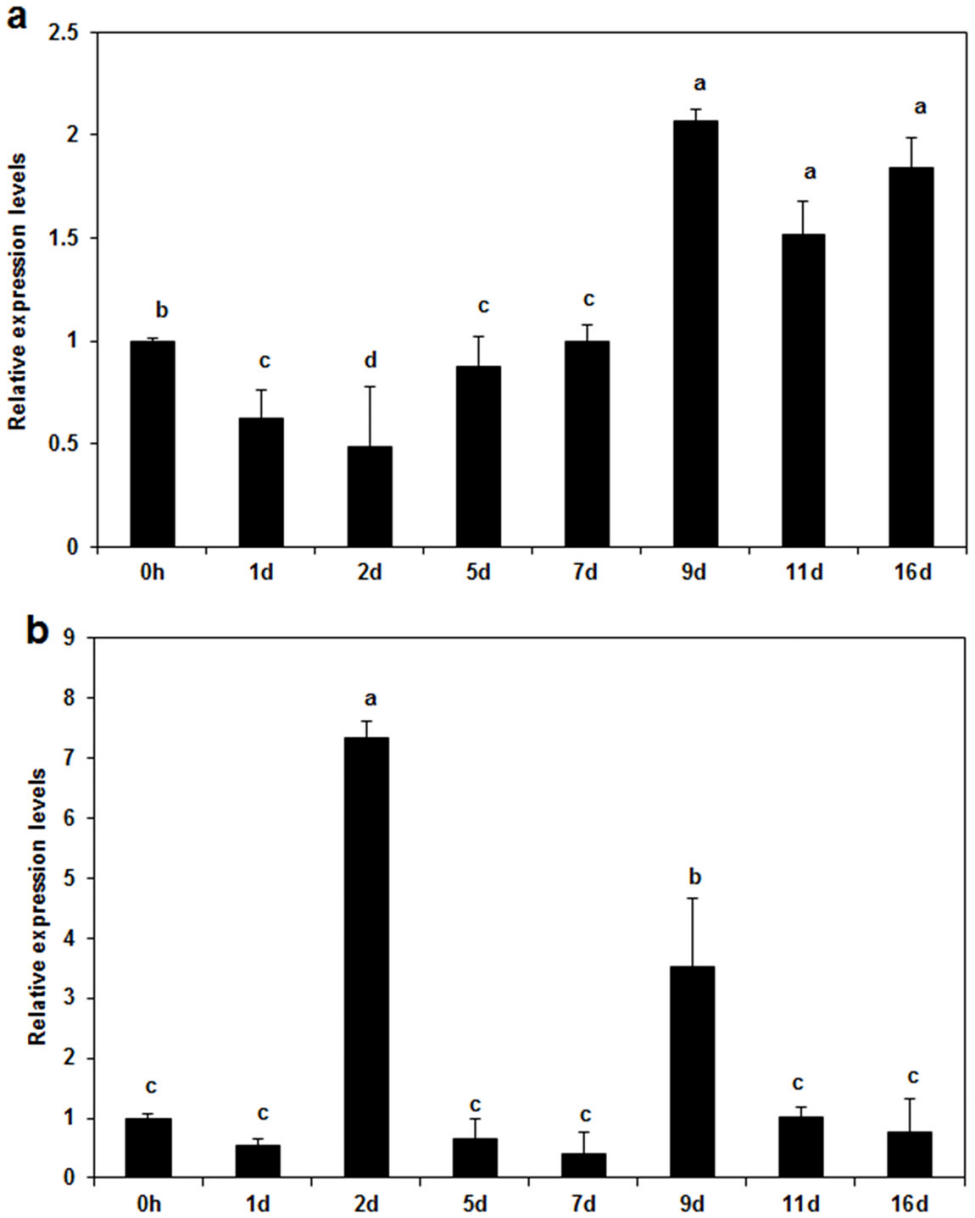
Effects of Hhs.015 fermentation broth on expression levels of *CALS1* and *CALS2* using EF1 as a reference gene. Gene expression levels of *CALS1* and *CALS2* were normalized to that of *EF1* (M&M). a: *CALS1* and b:*CALS2*. Bars represent the standard deviations. Each sample was amplified three times. Statistical significance of the differences between means was evaluated using One-WayANOVA. Different letters denoted significant differences at *p*<0.05 for time factor.

Next, the effects of Hhs.015 on the relative gene expression levels of host resistance-related *CALS1* and *CALS2* were investigated. For *CALS1*, gene expression level initially decreased after treatment, then rebound and subsequently increased (Fig 6a, *p*<0.05 for time factor). For *CALS2*, gene expression level remained low throughout the experiment except at 2 and 9 d post-treatment (Fig 6b, *p*<0.05 for time factor).

## Discussion

A previous study has shown that *Saccharothrix yanglingensis* Hhs.015 can suppress apple Valsa canker in both laboratory antagonism tests and field trials [28]. However, the molecular mechanisms underlying the effects of Hhs.015 remain largely unknown limiting its application on a wider scale.

In the present study, a specific RT-qPCR method was developed to examine the biocontrol mechanisms of Hhs.015 against *V*. *mali* from the perspectives of antifungal effect and host induced resistance. The result showed that Hhs.015 prevented *V*. *mali* infection by inhibiting pathogen growth, down-regulating pathogenicity factor expression, and inducing host resistance to the pathogen. This study provided new insight into the biocontrol mechanisms of Hhs.015 against apple Valsa canker.

Without excluding cross-reaction with other *Venturia* spp., a specific RT-qPCR assay was developed using targeting primers of internally transcribed spacer 2 regions of the 5.8S rRNA to accurately monitor the growth of a pure *V*. *inaequalis* isolate (a fungal pathogen that cause apple scab) in apple leaves in a controlled greenhouse environment [29]. All primers used in the present study were shown to be specific against their respective targets (S1 Fig). They were successfully used to quantify the pathogen, pathogenicity expression and host resistance factors on Fuji twigs in a controlled indoor environment. However, these primers have not been reported previously to be specific for other *Valsa* or *Malus* species. Thus, their specificity needs to be further verified with naturally infected samples from apple orchards. Such studies will help to avoid the possibility of cross-reaction with other species of *Valsa* and *Malus*.

DNA from dead cells can persist for a long period of time and therefore can still serve as a template for a PCR reaction even though cells may have died previously [30]. Moreover, plant DNA laddering can be induced by necrotrophic fungi, significantly affecting the quantification of pathogen and host resistance factors [15]. By contrast, mRNA is highly unstable having a short half-life [31] thus is a better marker for cell viability. In the present study, the RT-qPCR assay targeting *V*. *mali G6PDH* was highly sensitive with a minimum detection limit of 100 pg/μL cDNA (Fig 1). Thus, it appears ideally suited for the purpose of detecting and quantifying the apple Valsa canker present in apple tissues.

RT-qPCR data revealed that Hhs.015 treatment led to a drastic reduction of *V*. *mali* in infected but asymptomatic bark after an initial slight increase (Fig 3a). The initial pathogen increase suggested a burst of pathogen growth possibly stimulated by the nutrient supplements from the fermentation broth. The subsequent pathogen decrease indicated the presence of bioactive substances produced by Hhs.015 (e.g., antibiotics, and exoenzymes), which effectively inhibited cell growth or killed cells of the pathogen.

Hhs.015 treatment had no significant effect (*p*>0.05) on the amount of *V*. *mali* at the boundary between diseased and healthy bark tissues (Fig 3b) but significantly inhibited (*p*<0.05 for treat factor) the relative expansion rate of the lesion (Fig 4). Similarly, a previous study has demonstrated that *Cylindrocarpon* DNA concentrations in apple roots were not correlated with growth reduction observed in apple seedlings [32]. A possible mechanism in this situation may be that the pathogen pre-colonized the healthy host tissues and damaged host cell function by secreting cell wall degrading enzymes or toxins without changing the overall pathogen levels.

Additionally, it has been reported that the quantity of Candidatus *Phytoplasma mali* colonizing apple trees was invariant relative to changes in pathogenicity. This phenomenon might result from micro-variations in abiotic factors (e.g. temperature, light, and water supply) which can modulate the physiological conditions of plants and lead to varying resistant responses to pathogens [33]. In the present study, because the analysis of canker symptom development may depend more on qualitative than quantitative factors [34], the amount of *V*. *mali* present in boundary bark tissues is unlikely to be a good indicator of disease symptom severity.

Cell wall-degrading enzymes were shown to play a role in infection of apple by *V*. *mali*, and were responsible for the development of swollen, soft, water-soaked lesions. A variety of pectinases including pectinesterase, pectinlyase, protopectinase, and pectin hydrolase (polymethylgalacturonase and polygalacturonase) [35] have been shown to be a key pathogenic factor in fungi [4]. Thus, we examined the gene expression profile of *EPG*, one kind of pectinases, in apple bark tissue between diseased and healthy areas. The relative gene expression level of *EPG* at the bark tissue boundary decreased sharply at 9 d post treatment (Fig 5, *p*<0.05 for time factor), indicating that fungal pathogenicity was effectively controlled by Hhs.015. Our results were consistent with previous findings regarding plant-pathogen-biocontrol agent interactions in other species. For example, endo- and exo-polygalacturonase of *Botrytis cineres* were deactivated by *Trichoderma harzianum* isolates, leading to reduced disease incidence of grey mouldin bean leaves [36]. Moreover, *Pseudomonas fluorescens* strain KD has been shown to have biocontrol attributes against the phytopathogenic oomycete *Pythium ultimum*in cucumber by decreasing polygalacturonase activity in the pathogen [37].

Pathogens induce plants to generate resistant reactions through diverse physical and chemical processes such as lignification and suberization, accumulation of phenolics, hypersensitive response, generation of phytoalexin, and formation of pathogenesis-related proteins [38]. To affect physical resistance, plants can be induced to form a callose around the pathogen which is filled with phenolics on the inner wall of the host cell [39]. Deposition of callose is one of the first steps in host induced resistance against pathogens [40]. In the present study, the RT-qPCR assay showed that the relative gene expression levels of host resistance-related *CALS1* and *CALS2* increased at the bark tissue boundary especially at 9 d post-treatment (Fig 6, *p*<0.05 for time factor). The progressive increase in *CALS* gene expression coincided with a reduction in pathogenicity-related *EPG* gene expression (Fig 5). At 2 d post-treatment, Hhs.015 may have induced expression of the host resistance factor gene through physical (e.g. invasion) and/or chemical (e.g. antibiotic) pathways. At 9 d post treatment, the induced gene expression level of *CALS2* coincided with that of *CALS1* suggesting that Hhs.015 enhanced host resistance against *V*. *mali*. These changes may result from one or more of the following mechanism (s): 1) a direct effect of Hhs.015 inhibition on pathogen growth in the bark lesions, and/or 2) the indirect effect of a Hhs.015-stimulated increase in host resistance to the pathogen. A previous study has demonstrated that callose deposition in plasmodesmata was mainly responsible for restricting the movement of *Soybean mosaic virus* between plant cells contributing to the defensive response of soybeans to viral infection [14].

In the present study, co-analysis of the host cDNA was used to normalize the efficiency of total RNA extraction. On the one hand, the quantity of RNA isolated from plant tissue largely depends on how well the rigid plant cell walls are mechanically disrupted. On the other hand, in plant tissues, the quality of isolated RNA is affected by the presence of substantial secondary metabolites (e.g. polysaccharides and polyphenols), especially at infection sites [29]. Therefore, a cDNA calibration factor was necessary for the RT-qPCR assays of total RNA extracted from apple bark tissues in order to normalize the levels of pathogen detected.

In conclusion, this study has developed a *V*. *mali* sensitive RT-qPCR method for simultaneously quantifying pathogen levels and monitoring pathogenicity gene expression and host resistance factor in apple Valsa canker. The results provide new insights into the molecular mechanisms of canker control by *Saccharothrix yanglingensis* Hhs.015 based on its antifungal activity and stimulation of host induced resistance. Hhs.015 prevents infection of apple twigs by *V*. *mali* through multiple mechanisms, such as inhibiting pathogen growth, down-regulating pathogenicity factor expression, and inducing a high level either direct or indirect host resistance. To further understand the relevant mechanisms in this system, it is crucial to identify what types of bioactive substances, if any, play a role in plant-pathogen-biocontrol agent interaction. The developed RT-qPCR approach is sensitive and reliable, thus providing a useful tool for disease management, breeding programs, and plant-pathogen interaction studies of apple Valsa canker. The availability of such a method will eventually help mitigate the threat of this disease to fruit production in apple producing countries of the world.

## Supporting Information

S1 Fig Agarose gel electrophoresis of PCR products obtained using gene-specific primer. Lanes 1 and 4: PCR products obtained using *Valsa mali* cDNA as a template along with the primers for *G6PDH* and *EPG*, respectively; lanes 2 and 5: healthy bark smeared with fermentation broth of *Saccharothrix yanglingensis* Hhs.015 only; lanes 3 and 6: control; lanes 7, 10 and 13: PCR products obtained using the cDNA of healthy bark smeared with Hhs.015 fermentation broth as a template along with the primers of *EF1*, *CALS1* and *CALS2*, respectively; lanes 8, 11 and 14: *V*. *mali*; and lanes 9, 12 and 15: control; M: marker 2000.
(TIF)
