# Supplementary figures and images for: Study on Interactions between the Major Apple Valsa Canker Pathogen Valsa mali and Its Biocontrol Agent Saccharothrix yanglingensis Hhs.015 Using RT-qPCR
Source: PLoS One. 2016 Sep 9;11(9):e0162174. doi: 10.1371/journal.pone.0162174 (PMC5017705; doi:10.1371/journal.pone.0162174)

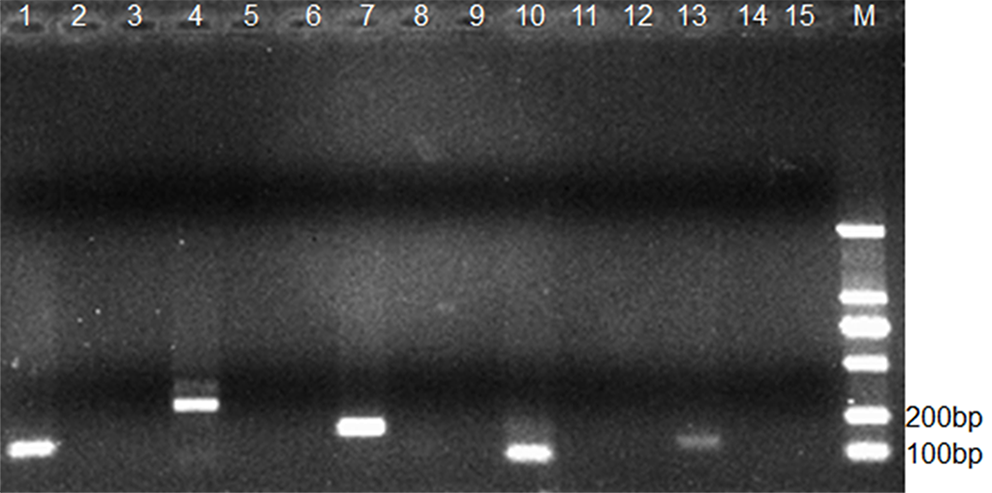

Supplement: S1 Fig — Lanes 1 and 4: PCR products obtained using Valsa mali cDNA as a template along with the primers for G6PDH and EPG, respectively; lanes 2 and 5: healthy bark smeared with fermentation broth of Saccharothrix yanglingensis Hhs.015 only; lanes 3 and 6: control; lanes 7, 10 and 13: PCR products obtained using the cDNA of healthy bark smeared with Hhs.015 fermentation broth as a template along with the primers of EF1, CALS1 and CALS2, respectively; lanes 8, 11 and 14: V. mali; and lanes 9, 12 and 15: control; M: marker 2000. (TIF) [file pone.0162174.s001.tif]
